# Point-of-Care Capillary Blood Creatinine: A Prospective study in Cardiology and Nephrology Outpatients

**DOI:** 10.26502/fccm.92920229

**Published:** 2021-12-03

**Authors:** Eve Vilaine, Paul Gabarre, Alain Beauchet, Alexandre Seidowsky, Olivier Auzel, Marie Hauguel-Moreau, Olivier Dubourg, Nicolas Mansencal, Marie Essig, Ziad A. Massy

**Affiliations:** 1 Service de néphrologie-hémodialyse, hôpital Ambroise Paré, AP-HP, 9 avenue du Général de Gaulle, F-92104 Boulogne-Billancourt, France.; 2 Service de néphrologie-hémodialyse, clinique Turin, 3 rue de Turin, F-75008 Paris, France.; 3 Département épidémiologie-santé publique, hôpital Ambroise Paré, AP-HP, 9 avenue du Général de Gaulle, F-92104 Boulogne-Billancourt, France.; 4 Service de néphrologie-hémodialyse, hôpital Américain de Paris, 63 boulevard Victor Hugo, F-92200 Neuilly-sur-Seine, France.; 5 Service de cardiologie, hôpital Ambroise Paré, AP - HP, 9 avenue du Général de Gaulle, F-92104 Boulogne-Billancourt, France. Centre de référence des Maladies Cardiaques Héréditaires, Université de Versailles-Saint Quentin (UVSQ), Boulogne-Billancourt, France.; 6 Service de cardiologie, hôpital privé de Parly II, 21, rue moxouris, 78150 Le Chesnay; 7 Inserm U1018, Équipe 5 CESP et UVSQ, Paris Saclay, 16 avenue Paul Vaillant-Couturier, F-94800 Villejuif, France.

**Keywords:** Capillary blood, contrast-induced nephropathy, Nephroprotection, Point-of-care blood creatinine test, POC

## Abstract

**Background:**

The radiological or interventional use of contrast medium exposes patients to a risk of contrast-induced nephropathy. Pre-existing kidney failure is a major risk factor. Point-of-Care Capillary blood creatinine tests are promising; their speed might help to optimize treatment decisions and patient care in these situations.

**Methods:**

The objective of the present study was to assess the ability of a new point of care capillary blood creatinine test (Stat Sensor X-press, Nova Biomedical Cooperation, Waltham, MA, USA) to diagnose kidney failure, relative to a standard lab-based plasma creatinine assay. A total of 113 patients 33 women (29.2%) were included. The capillary blood creatinine concentration was significantly correlated with the plasma creatinine concentration in both men (Pearson’s r [95% Confidence Interval (CI)] = 0.84 [0.75 – 0.89]; p<0.001) and women (Pearson’s r [95%CI] = 0.95 [0.89 – 0.97]; p<0.001). The test’s diagnostic performance was satisfactory, its sensitivity was 70% [35 – 93] in women and 78% [52 – 94] in men, and its specificity was 91% [72 – 99] in woman and 93% [84 – 98] in men.

**Conclusion:**

Rapid Point-of Care Capillary creatinine test is an easy-to-use, accurate tool for detecting kidney failure before a patient is exposed to procedures involving contrast medium. The POC test performed less well in patients over the age of 75 and in patients with high plasma creatinine level

## 1. Background

The use of iodinated contrast agents in radiological examinations and interventional procedures (e.g. coronary angiography) is major: in 2003, 8 million litres of these compounds were used worldwide in 80 million procedures [1]. The use of iodinated contrast agents exposes the patient to a risk of Contrast-Induced Nephropathy (CIN), which is one of the most frequent types of in-hospital acute kidney injury [2]. The consensus definition of CIN is an absolute increase in plasma creatinine concentration from baseline of at least 44 μmol/l or a relative increase of at least >25% within 72 h of the intravascular injection of iodinated contrast agents, in the absence of any identifiable alternative cause for the increase [3]. The occurrence of prolonged kidney injury after coronary angiography is associated with an elevated long-term risk of mortality [4,5] and renal replacement therapy. Contrast-induced nephropathy also prolongs the length of hospital stay [6].

The prevention of CIN is mainly based on the removal of modifiable risk factors (renal hypoperfusion, anaemia, the concomitant use of nephrotoxic drugs, etc.) and the identification of high-risk patients (age>75, diabetes, chronic renal disease, myeloma, heart failure, etc.) [7]. To this end, several scores for predicting the occurrence of CIN have been developed in the last few years [8]. For example, Duan et al recently described a pre-procedure risk score for contrast-induced acute kidney injury that took account of five variables: age, plasma levels of creatinine (in μmol/l), N-terminal pro B-type natriuretic peptide and ultrasensitive C-reactive protein, and primary percutaneous coronary intervention. The score was used to classify patients’ level of risk as low, moderate, high or very high with a good degree of discrimination (C statistic [95% Confidence Interval (CI)] = 0.809 [0.749–0.870]) and a higher predictive value than for previously validated scores [9]. Duan et al. found that pre-existing kidney failure was the main risk factor for CIN. In an emergency setting, the turnaround time for conventional laboratory assays remains a key issue; a bedside diagnosis of kidney failure is not possible. Rapid Point-of-Care (POC) creatinine tests might be able to solve this time issue. The tests are easy to use and require a sample of capillary blood, which is less burdensome than the collection of a venous or arterial blood sample. The result is given between 30 seconds and 8.5 minutes later (depending on the device), vs. 1 to 2 hours for laboratory assays [10]. Treatment and care decisions can therefore be taken more rapidly [11]. However, few studies have assessed the ability of POC capillary blood creatinine tests to screen for kidney failure. Hence, the objective of the present study was to evaluate the ability of a POC capillary blood creatinine test to diagnose kidney failure, versus a conventional lab-based plasma creatinine assay in cardiology and nephrology outpatients.

## 2. Methods

### 2.1. Study population and objectives

We performed a prospective, single-centre study of routine clinical practice in the cardionephrology outpatient unit at Ambroise Paré University Hospital (Boulogne-Billancourt, France). Between October 2015 and June 2016, we included all consecutive patients aged 18 or over attending the outpatient unit for diagnostic and/or interventional procedures involving the injection of iodinated contrast agent (contrast-enhanced computed tomography, peripheral arteriography, coronary angiography alone, or coronary angiography combined with percutaneous angioplasty) or for nephrological or cardiovascular examinations not involving the use of iodinated contrast agent. There were no exclusion criteria. The study’s primary objective was to evaluate the diagnosis of kidney failure on the basis of the capillary blood creatinine level (Ccap) versus the plasma creatinine level (Cpl) in consecutive patients attending the cardionephrology outpatient unit. The study’s secondary objective was to evaluate the diagnosis of kidney failure on the basis of the glomerular filtration rate calculated from the Ccap (eGFRcap) versus that calculated from the Cpl (eGFRpl) in the same patients.

### 2.2. Assay methods

The Ccap was measured with a POC test (Stat Sensor X-press, Nova Biomedical Corporation, Waltham, MA, USA) used routinely in the outpatient unit. After the patient had fasted overnight, a nurse collected a drop of capillary blood from the finger, placed it on the device’s test strip, and inserted the strip into the reader. The result (expressed in μmol/l) was given 25 seconds later. The assay method is based on the transformation of creatinine into hydrogen peroxide, as catalysed by three enzymes in the following reactions:


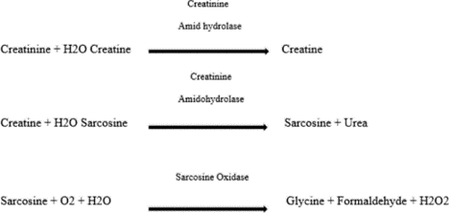


The hydrogen peroxide was then detected electrochemically; the redox reaction generates a current that is directly proportional to the creatinine concentration in the sample [12]. Although there are no analytical standards for POC tests, the National Kidney Disease Education Program’s Laboratory Working Group has stated that the measurement imprecision should be less than 8% (relative to calibration with isotope dilution mass spectroscopy (IDMS)) and the bias should be less than <5% for a Cpl above 88.4 μmol/L [13]. The device used in the present study met these criteria. The eGFRcap was estimated from the Ccap, using the Chronic Kidney Disease–Epidemiology Collaboration (CKD-EPI) equation [14]. In parallel, the Cpl was measured in a two-point, kinetic enzyme assay (VITROS^®^ CREA, Ortho Clinical Diagnostics, Raritan, NJ, USA that had been calibrated with IDMS. The fasted patient’s blood sample was collected by venepuncture of the arm with a 22 gauge needle and sent to the hospital’s central laboratory. The laboratory estimated the eGFRpl from the plasma creatinine level, using the CKD-EPI equation.

### 2.3. Statistical analysis

The thresholds for kidney failure were a creatinine level of 110 μmol/l for men and 96 μmol/l for women, and an eGFR below 60 ml/min/1.73m^2^, according to the CKD-EPI equation. Quantitative variables were expressed as the mean ± standard deviation (SD), and qualitative variables were expressed as the frequency (percentage). The degree of correlation between the capillary blood measurements (Ccap and eGFRcap) and the plasma measurements (Cpl and eGFRpl) was assessed by calculation of Pearson’s correlation coefficient (r). The level of agreement between the capillary and plasma measurements was evaluated by calculation of the intraclass correlation coefficient (ICC) and graphically in a Bland-Altman plot.

The performance indicators (Sensitivity (Se) and Specificity (Sp)) for the diagnosis of kidney failure on the basis of the capillary blood measurements (Ccap and eGFR cap) with the Stat Sensor X-press versus the plasma measurements (Cpl and eGFRpl) were calculated, and a receiver operating characteristic ROC curve was used to determine the eGFRcap threshold for a diagnosis of kidney failure. All statistical analyses were performed with R Core Team (version 3.2.3. 2013). R a language and environment for Statistical computing. R Foundation for Statistical Computing, Vienn, Austria. URL http://www.R-project.org.

## 3. Results

### 3.1. Characteristics of the study population

A total of 113 patients (33 women (29.2%)) were included in the study. The median age was 67, and the mean ± SD age was 64.8 ± 14.4 overall, 64.9 ±17.1 for the women, and 64.8 ± 13.2 for the men. Thirty-one patients (27.4%) were over 75. The general characteristics of the study population are summarized in Table 1. We noted that 45.1% of the patients had undergone coronary angiography, 1.8% had undergone coronary angiography with angioplasty, 22.1% had undergone angioscopy, and 1.8% had undergone peripheral arteriography. The kidney-related characteristics of the study population (Ccap, Cpl, eGFRcap, and eGFRpl) are summarized in (Table 2). The eGFRpl was above 60 ml/min/1.73m^2^ in 79 patients (69.9%), between 30 and 60 ml/min/1.73m2 in 24 (21.2%), between 15 and 30 ml/min/1.73m^2^ in 6 (5.3%), and below 15 ml/min/1.73m^2^ in 4 (3.6%).

### 3.2. Correlation and level of agreement between capillary and plasma creatinine measurements

**Figure 1:**
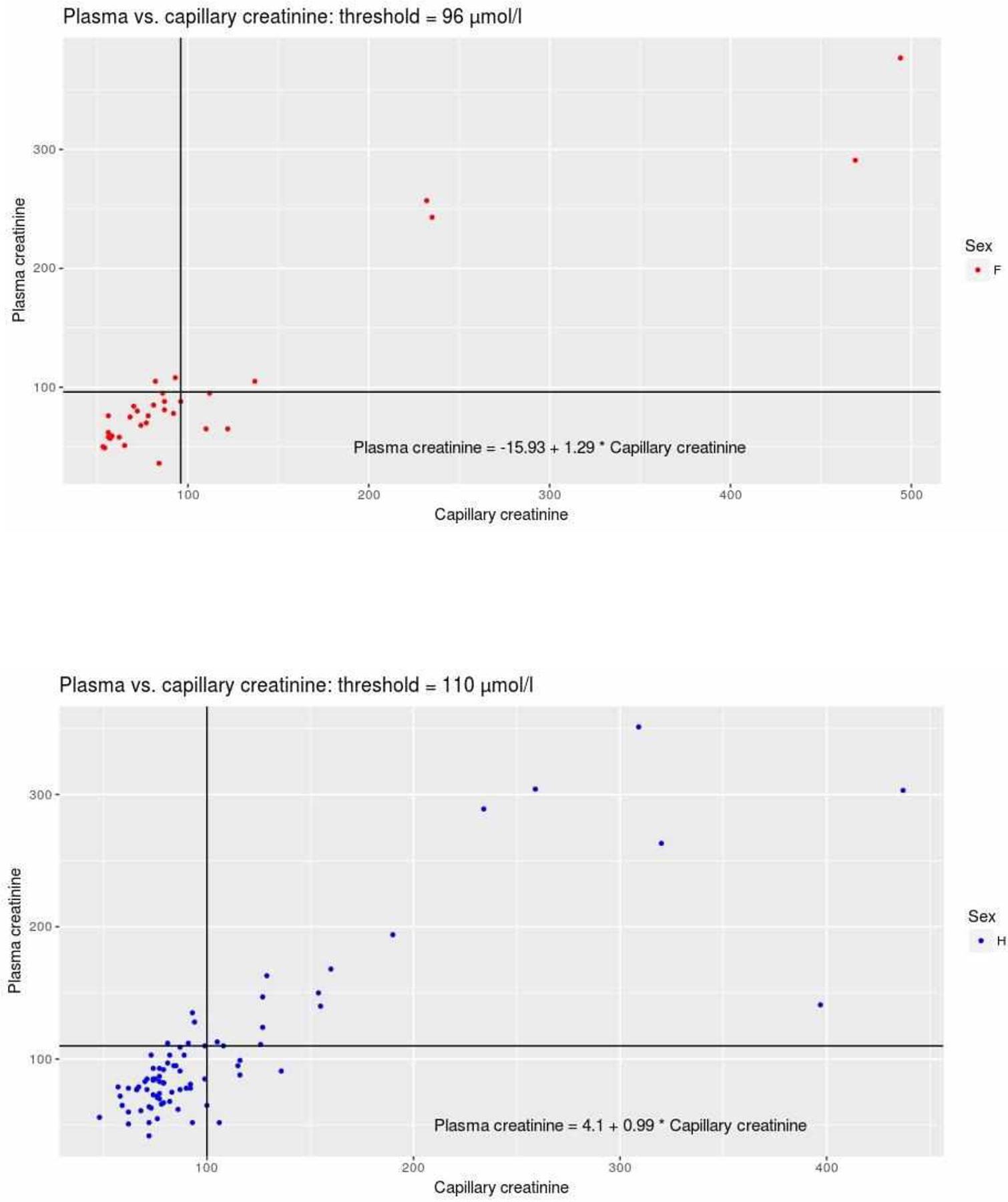
A. Correlation between Ccap(μmol/l) and Cpl(μmol/l) in women(n=30). The horizontal and vertical lines represent the threshold for definition of the true positive (i.e. 96 μmol/L for women). B. Correlation between Ccap(μmol/l) and Cpl(μmol/l) in men (n=80). The horizontal and vertical lines represent the threshold for definition of the true positive (i.e. 110 μmol/l for men).

**Figure 2:**
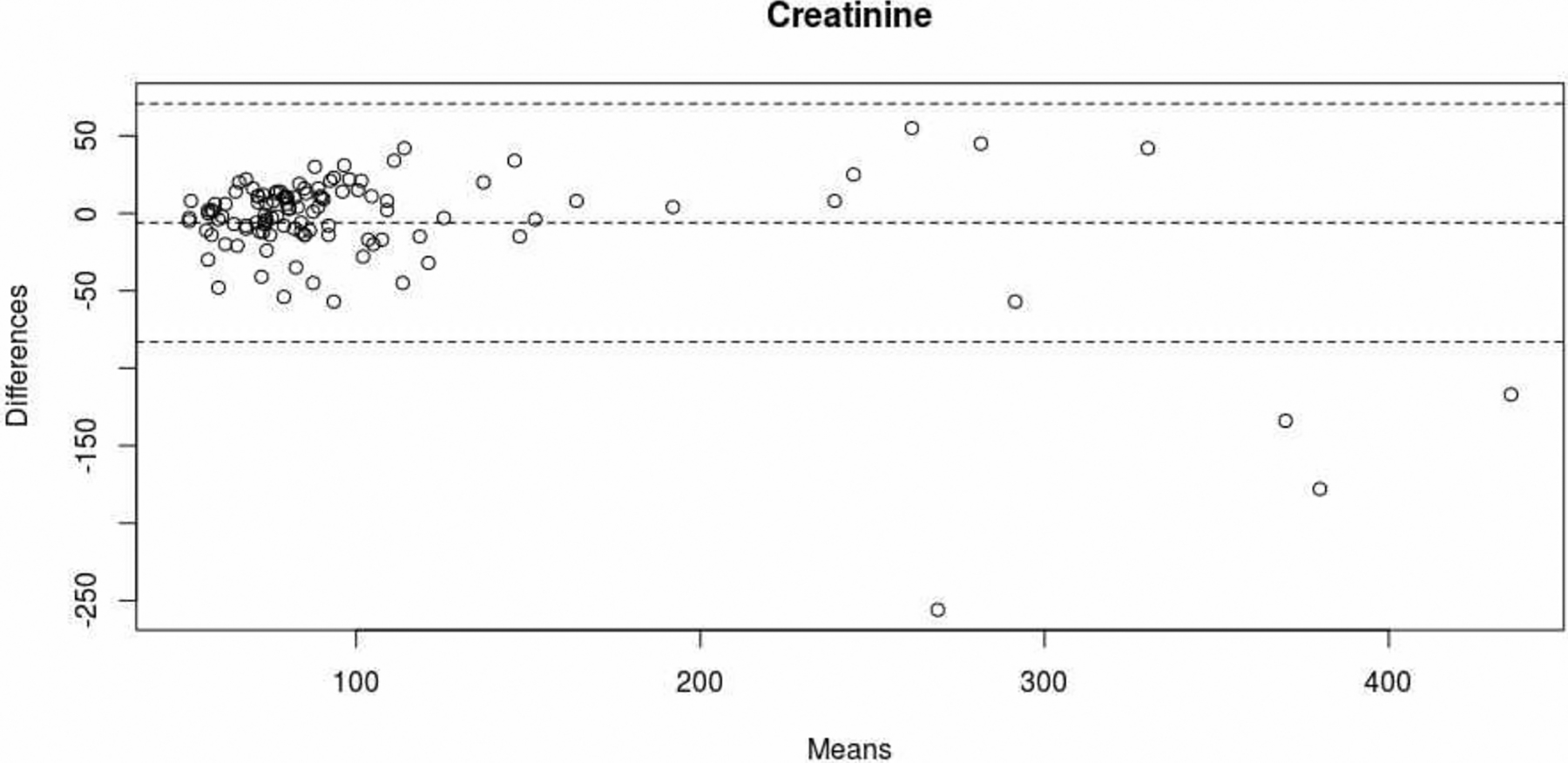
Level of agreement between the Ccap and Cpl measurements (Bland-Altman plot). The dotted lines correspond to the mean [95%CI] for the difference between Ccap and Cpl.

Ccap was significantly correlated with Cpl in both men (Pearson’s r [95%CI] = 0.84 [0.75 – 0.89]; p<0.001) and women (r [95%CI] = 0.95 [0.89 – 0.97]; p< 0.001) (Figure 1). Furthermore, there was a very good level of agreement between Ccap and Cpl; the ICC [95%CI] was 0.92 [0.85 – 0.96] in men and 0.99 [0.93–1] in women. The mean difference [95%CI] between Ccap and Cpl was −6.0 μmol/L [−82.9 – 70.8] (Figure 2). The degree of dispersion was large - particularly for high values. In the four patients with a Cpl above 390 μmol/L (two men aged 67 and 91, and two women aged 69 and 79), the mean difference between Ccap and Cpl was −171 μmol/L. In the patients with Ccap and Cpl values below the threshold (n=77), the mean difference [95%CI] was −0.8 μmol/L [−31.9 – 30.3].

### 3.3. Correlation and level of agreement between capillary and plasma eGFR values

**Figure 3:**
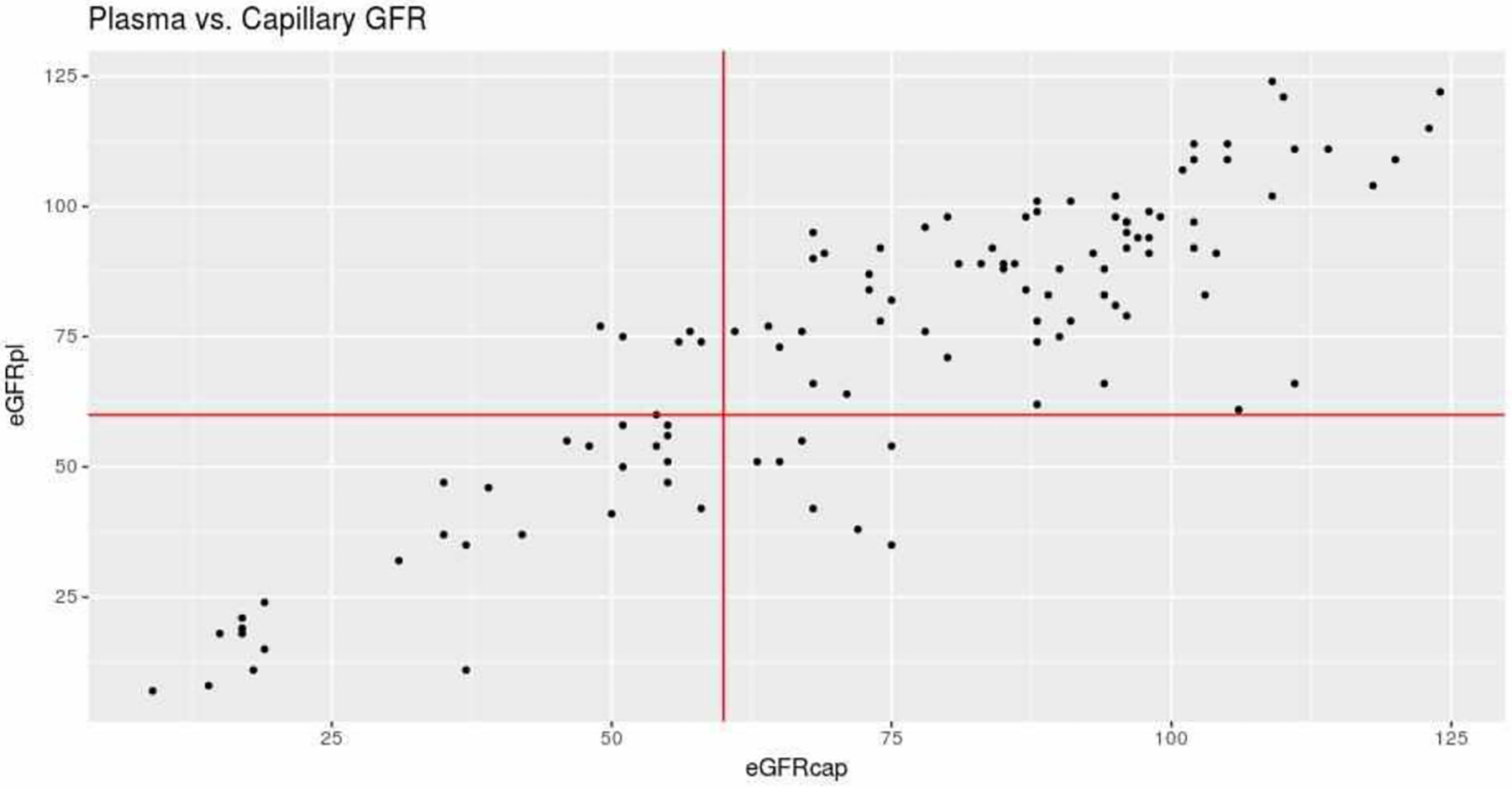
Correlation between eGFRcap and eGFRpl (in ml/min/1.73m^2^). The vertical and horizontal red lines correspond to the threshold for the definition of kidney failure in each of the estimations (i.e. 60 ml/min/1.73m^2^).

**Figure 4:**
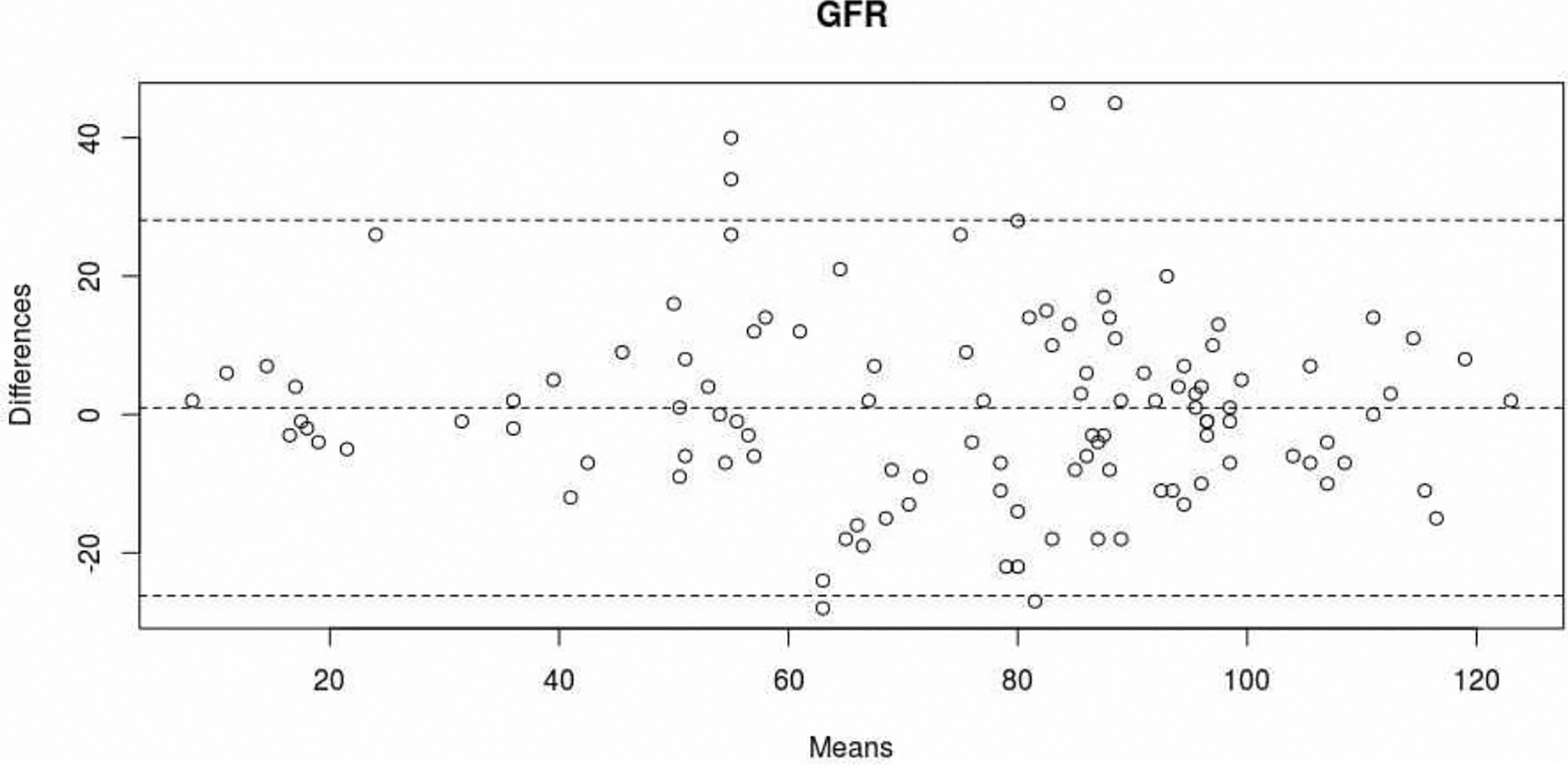
Level of agreement between eGFRcap and eGFRpl (Bland-Altman plot). The dotted lines correspond to the mean [95%CI] for the difference between eGFRcap and eGFRpl).

There was a good degree of correlation between eGFRcap and eGFRpl (Pearson’s r [95%CI] = 0.88 [0.83 – 0.91]; p<0.001) (Figure 3). Likewise, there was a very good level of agreement between eGFRcap and eGFRpl (ICC [95%CI] = 0.82 [0.71 – 0.89]). The mean difference [95%CI] between eGFRcap and eGFRpl was −0.9 μmol/L [−28.1 – 26.2] (Figure 4).

### 3.4. Diagnostic performance of the capillary blood test

The sensitivity [95%CI] for diagnosis of kidney failure using Ccap was 70% [35 – 93] in women and 78% [52 – 94] in men. The specificity was 91% [72 – 99] in women and 93% [84 – 98] in men. The sensitivity for diagnosis of kidney failure using eGFRcap was 80% [62 – 91], and the specificity was 92% [84 – 97]. A receiver operating characteristic curve analysis showed that the eGFRcap threshold for kidney failure was 60 ml/min/1.73m^2^. This threshold gave a true positive rate of 80%, a false positive rate of 6.4%, and an area under the curve of 0.963. Seven patients were classified as false negatives (i.e. no kidney failure, even though their eGFRpl was below 60 ml/min/1.73m^2^), according to eGFRcap. Five of the seven were over the age of 75, and the other two were respectively 70 and 74 years old.

## 4. Discussion

The Stat Sensor X-press POC capillary blood creatinine test can be easily used to diagnose kidney failure at the patient’s bedside prior to an examination involving an injection of iodinated contrast agent. The diagnostic sensitivity and specificity were 70–78% and 91–93%, respectively for women and men. Our study population was representative of patients at a high risk of developing CIN because they were elderly and had cardiovascular risk factors. The median age was 67, and a third of the patients were over 75. The performance of capillary creatinine measurements has been evaluated in several studies, the main results of which are summarized in Table 3. An Australian study evaluated the performance of the Nova StatSensor as a screening tool for chronic kidney failure in 100 individuals (63 patients with kidney failure and 37 healthy volunteers), in whom Ccap ranged from 46 to 962 μmol/l. The level of agreement between Ccap and Cpl was lower at higher concentrations. Hence, 18% of the creatinine concentration below 150 μmol/l differed by more than 20 μmol/l from the lab value, whereas the proportion for concentrations above 150 μmo/l was 62% [15]. The measurement imprecision for plasma creatinine values below 150 μmol/l was 8.9%.

The Nova StatSensor’s analytical performances have also been evaluated in a Canadian study (Table 3) [16]. The coefficient of variation for Ccap ranged from 4.5% to 9.1% for values between 93 and 863 μmol/l. The capillary and plasma measurements were closely correlated (R^2^=0.9328); again, however, there was negative bias (−30%) at higher creatinine values. The SD of the estimation was 20 μmol/l for Cpl values between 100 and 200 μmol/l. Our results are in line with these studies, i.e. a close correlation between Ccap and Cpl but notable bias at higher values of Cpl. However, the patients in whom notable bias was identified all had a Ccap value that enabled an unambiguous diagnosis of kidney failure and thus the adoption of the measures to prevent CIN. The significant bias in patients with late-stage kidney failure might be due (at least in part) to analytical interference by high blood concentrations of urea. In fact, urea is an intermediate product of the enzyme reaction used in the POC test device and inhibits the hydrolysis of creatinine into sarcosine and urea.

In patients under the age of 75, the ability of Ccap and eGFRcap to diagnosing kidney failure prior to the performance of diagnostic and/or therapeutic procedures involving an iodinated contrast agent appears to be acceptable. In patients aged 75 and over, the diagnostic performance of Ccap or eGFRcap appears to be less good. However, this point should be confirmed in additional studies of a larger number of elderly participants; our sample size in this age class was low.

## 5. Conclusion

Our study confirmed the value of a POC capillary blood creatinine test for diagnosing (at least in patients under the age of 75) kidney failure prior to the injection of iodinated contrast agent in an emergency setting – i.e. when the turnaround time for a lab assay is too long. The rapid, simple, reliable identification of patients with impaired renal function may have a significant impact in daily practice by enabling the implementation of preventive measures and thus limiting the risk of CIN. POC capillary blood creatinine test for is promising because it is an easy-to-use, accurate tool that gives quick results. Further studies in elderly populations are warranted because the false positive rate in this age class is still too high.

## Figures and Tables

**Table 1:** Baseline characteristics.

Patients (n=113)	
Sex, male (%)	80 (71%)
Age, years	64.8 ±14.4
Body mass index, kg/m^2^	26.1 ±6.8
Chronic kidney disease	36 (32%)
Stage	
1	2 (5.5%)
2	5 (13.9%)
3	19 (52.8%)
4	4 (11.1%)
5	4 (11.1%)
5- Dialysis	2 (5.5%)
Anaemia	13/110 (11.8%)
History of heart failure	16/110 (11.8%)
LVEF§	55.2 % ±15.9
High blood pressure	59/110 (53.6%)
Diabetes mellitus	26/110 (23.6%)
Active smocking	31/105 (29.5%)
Dyslipidaemia	32/110 (29.1%)
NSAID	3 (2.7%)
ACE inhibitor/ARB	62 (56.4%)
Diuretics	27 (24.5%)
Haemoglobin£, g/dL	13.5 ±1.73
Test	
None	33 (29.2%)
Coronary angiogram	51 (45.1%)
Coronary angioplasty	2 (1.8%)
Angiography	25 (22.1%)
Peripheral angiogram	2 (1.8%)

LVEF: left ventricular ejection fraction; NSAID: non-steroidal anti-inflammatory drug; ACE: angiotensin-converting enzyme; ARB: angiotensin receptor blocker § data available for 67 patients £data available for 95 patients.

**Table 2:** Kidney-related characteristics of the study population (80 men and 33 women).

Cpl				CCap			eGFRpl			eGFRcap	
	Total	F	M	Total	F	M	Total	F	M	Total F	M
Mean	108.2	112.5	106.4	102.1	99.8	103.1	73.9	65.7	77.2	74.8 69.2	77.1
SE	7.7	18.2	7.9	6.1	13.4	6.6	2.7	5.2	3.1	2.6 5.1	3
Median	82	81	82	83	76	85	78	66	83.5	80 68	85.5
Min	48	53	48	36	36	42	7	7	11	9 9	15
Max	494	494	437	377	377	351	124	122	124	124 124	123

Cpl: plasma creatinine concentration; Ccap: capillary blood creatinine concentration; eGFRpl: eGFR estimated using the plasma creatinine concentration; eGFRcap: eGFR estimated using the capillary blood creatinine; SE: standard error; F: females; M: males.

**Table 3:** Studies having evaluated the performance criteria of the Stat Sensor X-press.

First author (year)	N (patients)	CCap range (μmol/l)	Creatinine threshold (μmol/l) for the diagnosis of kidney failure	Sensitivity (%)	Specificity (%)	Imprecision (%)
Shepard14 (2010)	100	46–962	150	96.2	78.7	7.8
Schnabl15 (2010)	NR	93–863	± 27	NR	NR	4.5–7
The present study	113	42–377	110 (males)	78	93	
			96 (females)	70	91	

## Data Availability

The datasets used and/or analysed during the current study are available from the corresponding author on reasonable request.

## Abbreviations:

CIN: Contrast induced nephropathy
POC: Point of Care
CCAP: capillary blood creatinine
CPL: plasma creatinine level
eGFRcap: Estimated Glomerular Filtration Rate calculated from the Ccap
eGFRpl: Estimated Glomerular Filtration Rate calculated from the Cpl
IDMS: Isotope Dilution Mass Spectroscopy
CKD-EPI: Chronic Kidney Disease–Epidemiology Collaboration
ICC: Intraclass Correlation Coefficient
Se: Sensitivity
Sp: Specificity

## References

[1] Katzberg RW, Haller C. Contrast-induced nephrotoxicity: clinical landscape. Kidney Int Suppl. (2006): S3- 7. Levey AS, Stevens LA, Schmid CH, Zhang YL, Castro AF, Feldman HI, et al. CKD-EPI (Chronic Kidney Disease Epidemiology Collaboration). A new equation to estimate glomerular filtration rate. Ann Intern Med 150 (2009): 604–612. 10.7326/0003-4819-150-9-200905050-00006 PMC2763564 19414839

[2] Nash K, Hafeez A, Hou S. Hospital-acquired renal insufficiency. Am J Kidney Dis 39 (2002): 930–936. 11979336 10.1053/ajkd.2002.32766

[3] Morcos SK, Thomsen HS, Webb JA. Contrast-media-induced nephrotoxicity: a consensus report. Contrast Media Safety Committee, European Society of Urogenital Radiology (ESUR). Eur Radiol 9 (1999): 1602–1613. 10525875 10.1007/s003300050894

[4] Abe M, Morimoto T, Nakagawa Y, Furukawa Y, Ono K, Kato T, Impact of Transient or Persistent Contrast-induced Nephropathy on Long-term Mortality After Elective Percutaneous Coronary Intervention. Am J Cardiol 120 (2017): 2146–2153. 29106836 10.1016/j.amjcard.2017.08.036

[5] Marenzi G, Lauri G, Assanelli E, Campodonico J, De Metrio M, Marana I, Contrast-induced nephropathy in patients undergoing primary angioplasty for acute myocardial infarction. J Am Coll Cardiol 44 (2004): 1780–1785. 15519007 10.1016/j.jacc.2004.07.043

[6] Sedhai YR, Golamari R, Timalsina S, Basnyat S, Koirala A, Asija A, Contrast-Induced Nephropathy After Cardiac Catheterization: Culprits, Consequences and Predictors. Am J Med Sci 354 (2017): 462–466. 29173356 10.1016/j.amjms.2017.05.010

[7] Ozkok S, Ozkok A. Contrast-induced acute kidney injury: A review of practical points. World J Nephrol 6 (2017): 86–99. 28540198 10.5527/wjn.v6.i3.86 PMC5424439

[8] Mehran R, Aymong ED, Nikolsky E, Lasic Z, Iakovou I, Fahy M, A simple risk score for prediction of contrast-induced nephropathy after percutaneous coronary intervention: development and initial validation. J Am Coll Cardiol 44 (2004): 1393–1399. 15464318 10.1016/j.jacc.2004.06.068

[9] Duan C, Cao Y, Liu Y, Zhou L, Ping K, Tan MT, A New Preprocedure Risk Score for Predicting Contrast-Induced Acute Kidney Injury. Can J Cardiol 33 (2017): 714–723. 28392272 10.1016/j.cjca.2017.01.015

[10] Lee EJ, Shin SD, Song KJ, Kim SC, Cho JS, Lee SC, A point-of-care chemistry test for reduction of turnaround and clinical decision time. Am J Emerg Med 29 (2011):489–495. 20825817 10.1016/j.ajem.2009.11.020

[11] Nichols JH, Kickler TS, Dyer KL, Humbertson SK, Cooper PC, Maughan WL, Clinical outcomes of point-of-care testing in the interventional radiology and invasive cardiology setting. Clin Chem 46 (2000): 543–550. 10759479

[12] Kosack CS, de Kieviet W, Bayrak K, Milovic A, Page AL. Evaluation of the Nova StatSensor® Xpress(TM) Creatinine point-of-care handheld analyzer. PLoS ONE 10 (2015): e0122433. 25886375 10.1371/journal.pone.0122433 PMC4401790

[13] Myers GL, Miller WG, Coresh J, Fleming J, Greenberg N, Greene T, National Kidney Disease Education Program Laboratory Working Group. Recommendations Page 9/14 for improving serum creatinine measurement: a report from the Laboratory Working Group of the National Kidney Disease Education Program. Clin Chem 52 (2006): 5–18. 16332993 10.1373/clinchem.2005.0525144

[14] Levey AS, Stevens LA, Schmid CH, Zhang YL, Castro AF, Feldman HI, CKD-EPI (Chronic Kidney Disease Epidemiology Collaboration). A new equation to estimate glomerular filtration rate. Ann Intern Med 150 (2009): 604–612. 19414839 10.7326/0003-4819-150-9-200905050-00006 PMC2763564

[15] Shephard M, Peake M, Corso O, Shephard A, Mazzachi B, Spaeth B, Assessment of the Nova StatSensor whole blood point-of-care creatinine analyzer for the measurement of kidney function in screening for chronic kidney disease. Clin Chem Lab Med 48 (2010): 1113–1119. 20482303 10.1515/CCLM.2010.238

[16] Schnabl KL, Bagherpoor S, Diker P, Cursio C, Dubois J, Yip PM. Evaluation of the analytical performance of the Nova StatSensor creatinine meter and reagent strip technology for whole blood testing. Clin Biochem 43 (2010): 1026–1029. 20416292 10.1016/j.clinbiochem.2010.04.055

